# Do dentists have better oral health compared to general population: a study on oral health status and oral health behavior in Kathmandu, Nepal

**DOI:** 10.1186/1472-6831-14-23

**Published:** 2014-03-22

**Authors:** Madhu Wagle, Tordis A Trovik, Purusotam Basnet, Ganesh Acharya

**Affiliations:** 1 Women’s Health and Perinatology Research Group, Department of Clinical Medicine, Faculty of Health Sciences, University of Tromsø - The Arctic University of Norway, Tromsø N – 9037, Norway; 2 Department of Clinical Dentistry, Faculty of Health Sciences, University of Tromsø - The Arctic University of Norway, Tromsø, Norway; 3 Department of Obstetrics and Gynecology, University Hospital of North Norway, Tromsø, Norway

**Keywords:** Dentist, Oral health behavior, Oral health status, Dental caries, DMFT, Periodontal status, CPITN

## Abstract

**Background:**

Dentists are considered role models by the general population in regards to oral hygiene and oral health behavior. This study aimed to access the oral health status of dentists and laypersons, and compare the dentists’ practice of preventive dentistry and oral self-care behaviors to that of the laypersons.

**Methods:**

This cross-sectional study recruited 472 participants (195 dentists and 277 laypersons from the general population). Their oral health/hygiene behavior was assessed using a standardized close-ended multiple choice questionnaire. Oral examination was performed to assess caries using Decayed Missed Filled teeth (DMFT) index and periodontal status using Community Periodontal Index of Treatment Needs (CPITN).

**Results:**

Ninety-six percent of dentists brushed their teeth at least once daily, using fluoridated toothpaste and 80.5% twice daily. Although 94% of laypersons brushed their teeth once daily, they seldom used fluoridated toothpaste. Ten percent of participants in each group were caries free. The mean number of teeth present in the oral cavity (27.4 versus 25.4), mean number of teeth with caries (1.8 versus 3.7) and fillings (2.5 versus 0.4) were significantly different (p < 0.0001) between dentists and laypersons, respectively. Regarding the periodontal status, 82% of dentists had CPITN score of 0 whereas 71% of laypersons had the highest score 3 (p = 0.007), and 81% of the laypersons reported tooth mobility compared to 1% of dentists (p < 0.0001).

**Conclusions:**

The participating dentists had better periodontal status and better self-reported oral health behaviors than the laypersons. Despite similar prevalence of caries in the two groups, the prevalence of decayed and unfilled teeth was lower among the dentists.

## Background

Good oral health is necessary for individual’s well-being and is integral to good general health [1]. Oral disease is one of the major public health problems worldwide since it can affect anyone regardless of age, gender, ethnicity or social status and is among one of the most expensive to treat [2]. Although there has been a marked improvement in dental health in many developed countries, the prevalence of oral diseases is still increasing in some developing countries [3]. Changes in dietary patterns, increased consumption of sweetened drinks, level of education [4], poor oral hygiene, smoking, and alcohol consumption [5] are the major contributors to the increased prevalence of oral diseases.

Dental caries and periodontal diseases are considered to be the major causes of global oral disease burden [1,6]. According to World Health Organization (WHO), worldwide 60-90% of school children and almost 100% of adults are suffering from dental caries and 15-20% of middle aged adults have severe periodontal diseases. Furthermore, higher rates of oral diseases are observed among the underprivileged groups of the society [5]. Most oral diseases are largely preventable; the challenge is to create appropriate conditions that will enable individuals and societies to enjoy good oral health [7,8].

Dentists and dental health professionals are educated to promote better oral hygiene in the society and their duty is to integrate preventive procedures as well as to motivate and educate their patients about the preventive oral health behaviors [9,10]. They are the role models for the patients [11] and are considered to have adequate knowledge to practice appropriate oral hygiene, dental care and oral health behaviour. We tested the null hypothesis that there is no difference in the oral health behavior and oral health status among the Nepalese dentists and the laypersons in general population. The aims of this study were: a) to assess and compare the oral health status among dentists and laypersons, b) to compare the dentists’ practice of preventive dentistry and oral self-care behaviors to that of the laypersons.

## Methods

The study was conducted during three months period from May-July in 2006. Among a total of 319 dentists registered with the Nepal Dental Association (NDA) 196 practicing in the capital city Kathmandu were invited to participate in this cross-sectional study, and 195 of them agreed. The dentists were contacted by telephone, informed about the study and an appointment was made according to their convenience.

**Figure 1:**
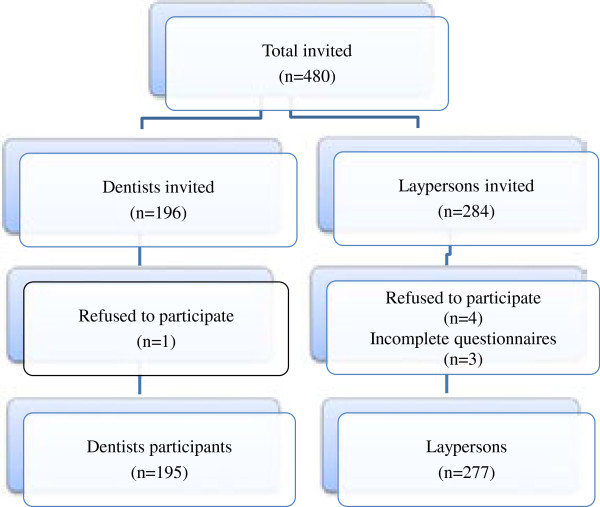
Flow chart of the study participants.

The laypersons (n = 284) were randomly selected among the sick patient accompanying persons; usually a family member, close relative or a friend, found in the waiting areas or canteens of hospitals. Among those 277 agreed to participate. At the time of this study there were 47 hospitals in Kathmandu valley including private and government hospitals. The two main hospitals, Bir Hospital and Tribhuvan University Teaching Hospital, were selected for the purpose as they offer treatment to both in-patients as well as to out-patients and they have the dental departments as a unit. Since both are government hospitals, representatives from different socio-economic groups visit for the treatment. Enrollment of participants is presented as a flow chart (Figure 1).

All study participants were informed about the study and those who agreed to participate signed a consent form. The dentist working outside the Kathmandu valley and healthy laypersons <20 years of age were excluded. An appointment, free of charge, was made for intra-oral examination for the participants who consented to the study. Ethical approval for conducting the study was obtained from the Nepal Health Research Council (NHRC) and the NDA. Approval for performing oral examination was obtained from the authorities of the respective hospitals and clinics.

The main tool for data collection was a set of questionnaire validated in a comparable target population [11] and an intra-oral examination to record the current oral health status. Decayed Missed Filled teeth (DMFT) index was used to assess dental caries and Community Periodontal Index of Treatment Needs (CPITN) was used to assess periodontal status. Participants filled in the questionnaires in a shielded office, before the clinical examination. The clinical examination for dentists was carried out in their own working place whereas the laypersons were examined at the dental unit of the respective hospitals under the same conditions. The examiner was blinded to the results of the questionnaire at the time of intra-oral examination.

The clinical data was collected while the patient was seated in a dental chair with professional light. A plain mouth mirror, a probe and a ball-tipped WHO dental explorer (CPI probe) were used to detect dental caries and periodontal pocket depths. The intra-oral examinations were performed by the one of the authors (MW) after calibration according to the WHO criteria for dental caries experience assessment [12] and periodontal status assessment [13] at the Department of Restorative Treatment and the Department of Periodontology at the Faculty of Dentistry, University of Bergen, Norway, before starting the study. Information about the oral health behavior was assessed by brushing habits, cleaning of interdental space, use of fluoridated toothpaste, and utilization of dental services.

All teeth except third molars were examined for the presence of dental caries on a dental chair with excellent light but without x-ray examination. The teeth examined for evaluating periodontal status were 17, 16, 11, 26, 27, 37, 36, 31, 46, and 47. Three indicators used to access the periodontal status were gingival bleeding, calculus, and periodontal pocket.

The diagnosis criteria and coding of the dental caries status used were according to the WHO standard [12], which are described below:

Code: Tooth condition

0 Sound crown (no evidence of treated or untreated caries)

1 Decayed crown (lesion in pit, fissure, or smooth tooth surface. Also temporarily filled or sealed but decayed teeth). Decayed teeth

3. Filled crown, with decay (one or more permanent restorations and one or more areas of decay. Filled root with decay

3 Filled crown, with no decay (one or more permanent restorations and no areas of decay). Filled root, with no decay

4 Missing tooth, as a result of caries

5 Permanent tooth missing, for any other reason

6 Fissure sealant

7 Bridge abutment, special crown or veneer

8 Unerupted crown (tooth space with an unerupted permanent tooth but without a primary tooth). Unexposed root

9 Not recorded (erupted permanent teeth that cannot be examined for any reason)

T Trauma (fracture) (missing surface due to trauma, no evidence of caries)

The criteria for diagnosis and coding for the periodontal status used were according to WHO and are as listed: healthy (code 0), bleeding on probing observed (code 1), calculus detected during probing (code 2), pocket 4 – 5 mm (code 3) and pocket >5 mm (code 4).

### Statistical methods

A priori sample size calculation was performed using the Raosoft Inc. Sample size calculator [14]. In Kathmandu, the number of NDA registered dentists at the time of survey was 319. With an assumption of an estimate of professional preventive knowledge being close to 50% and using an absolute precision of 0.05 and 95% confidence level the required number of participants were 175. To allow for possible refusals and drop-outs, we approached 196 dentists, of which 195 consented to the study.

The questionnaire contained four options for frequency of brushing for the dentists – once daily, twice daily, three times a day, and once a week; which during analysis was dichotomized to: brushing twice a day or more and once a day or less. There were six options for frequency of brushing teeth for the laypersons – not at all, once in a month or less, few times (3-4) a month, once a week, once daily, and two or more times a week. It was dichotomized into once a day and less during analysis. The question on interdental space cleaning had five possible answers ranging from cleaning after every meal to never. These were categorized into three groups (twice a day or more, once a day or less and never) for analysis. For the use of fluoridated toothpaste, there were five options – always or almost always to not at all, and an open space for the option if they use anything else. The variable was later dichotomized into always or almost always and seldom.

The study participants’ utilization of dental services was assessed by the duration of recent dental visit with six options- from no more than six months ago to never. It was categorized as – up to two years, more than two years, and never. Similarly, the reason for visit to the dental clinic, there were three options – had trouble with teeth/gums, regular checkup and other reasons. For analysis, it was further dichotomized to regular checkup and others.

All the data were coded and entered into a computer database. Data analysis was performed using SPSS 19.0 statistical package. Frequency tables for group comparison were processed and statistical evaluation was done using chi-square test for categorical variables and independent sample *t*-test for continuous parametric variables. A p-value of <0.05 was considered statistically significant.

## Results

A total of 472 participants completed the study. The male/female ratio was 71/124 among dentists and 199/78 among laypersons. Mean age of the dentists was 29.7 (SD 5.6) with age ranging from 24-56 years; whereas the laypersons had a mean age of 31.9 (SD 7.1) ranging from 20-53 years. All dentists and 38% of laypersons had at least bachelor level education. Seven percent of laypersons had no regular job where as only 0.5% of dentists reported to have no job.

The results of DMFT index are presented in Table 1. The prevalence of caries was 89.7% and 89.9% among dentists and laypersons, respectively. The prevalence of decayed teeth (DT) was 54.9% and 86.3%, reflecting the mean DT of 1.80 and 3.72, (p < 0.0001) and the prevalence of filled or restored teeth was 68.2% and 18.1%, reflecting the mean FT of 2.48 versus 0.39 (p < 0.0001), respectively among the dentists and the laypersons. Total DMFT was not significantly different between dentists and laypersons, but the difference was significant between males and females within both groups (p = 0.027 and p = 0.001, respectively). DT was significantly different between male and female laypersons (p = 0.003) but not the dentists (Table 1).

**Table 1:** Comparison of the DMFT (decayed, missed and filled teeth) index among dentists and laypersons

**Variables**	**Dentists (n = 195)**			**Laypersons (n = 277)**			p-value^*^
	**Male**	**Female**	**p-value**^**a**^	**Male**	**Female**	**p-value**^**b**^	
	**(n = 71)**	**(n = 124)**		**(n = 199)**	**(n = 78)**		
**DMFT**	3.85(3.4)	5.00(3.6)	0.027	4.08(3.0)	5.54(3.2)	0.001	
	**4.58(3.5)**			**4.49(3.1)**			**0.780**
DT	1.54(2.4)	1.95(2.3)	0.251	3.37(2.7)	4.60(3.1)	0.003	
	**1.80(2.4)**			**3.72(2.9)**			**<0.0001**
MT	0.31(0.9)	0.31(0.7)	0.980	0.34(0.7)	0.49(0.9)	0.228	
	**0.31(0.8)**			**0.38(0.8)**			**0.370**
FT	2.00(2.4)	2.75(3.0)	0.063	0.37(0.9)	0.45(1.2)	0.616	
	**2.48(2.8)**			**0.39(1.0)**			**<0.0001**

Data are expressed in mean (SD).

Bold numbers denote total mean (SD) and p-value among dentists and laypersons.

p-value^a^ : t-test for differences in mean between males and females among dentists.

p-value^b^ : t-test for differences in mean between males and females among laypersons.

p-value*: t-test for differences in mean among dentists and laypersons.

When comparing the CPITN score, self-reported gingival bleeding and tooth mobility, 82% of dentists had a CPITN score of 0, whereas 71% of laypersons had a highest CPITN score of 3 (p = 0.007). A statistically significant difference (p = 0.044) was found between male and female laypersons in relation to CPITN score 3.

Eighty-one percent of laypersons had self-reported tooth mobility compared to 1% of dentists (p < 0.0001). Similarly, 38% and 49% of the study participants among dentists and laypersons, respectively reported to have gingival bleeding (p = 0.018) and a statistically significant difference was found between male and female among the laypersons (p = 0.007) (Table 2).

**Table 2:** Comparison of the periodontal condition among dentists and laypersons using CPITN index, tooth mobility and gingival bleeding

**Variables**	**Dentists (n = 195)**			**Laypersons (n = 277)**			p-value^*^
	**Male**	**Female**	**p-value**^**a**^	**Male**	**Female**	**p-value**^**b**^	
	**(n = 71)**	**(n = 124)**		**(n = 199)**	**(n = 78)**		
**Periodontal status**							
CPITN Score 0	81.7	82.3	-	16.6	24.4	-	
	**82.1**			**18.7**			**<0.0001**
Score 2	5.6	2.4	0.233	5.0	3.8	0.672	
	**3.6**			**4.8**			**0.530**
Score 3	9.9	15.3	0.543	71.4	70.5	0.044	
	**13.3**			**70.7**			**0.007**
Score 4	2.8	-	-	7.0	1.3	0.875	
	**1.0**			**5.8**			**0.784**
**Tooth mobility**							
Yes	2.9	-	0.058	81.2	80.8	0.932	
	**1.0**			**81.1**			**<0.0001**
No	97.1	100.0		18.8	19.2		
	**99.0**			**18.9**			
**Gingival bleeding**							
Yes	36.6	63.4	0.826	43.6	61.8	0.007	
	**37.6**			**48.7**			**0.018**
No	61.8	38.2		56.4	38.2		
	**62.4**			**51.3**			

Data are expressed in percentage.

Bold numbers denote total participants percentage and p-value among dentists and laypersons.

p-value^a^ : *χ*^2^ test for differences between males and females among dentists.

p-value^b^ : *χ*^2^ test for differences between males and females among laypersons.

p-value*: *χ*^2^ test for differences in among dentists and laypersons.

Comparison of the number of teeth present in oral cavity among dentists and laypersons is presented in Table 3. The mean total number of teeth in the oral cavity was 27.35 among the dentists compared to 25.38 among the laypersons (p < 0.0001). The differences were also statistically significant between the males and females in both study groups. Similarly, there were significant gender-related differences with regards to the presence of teeth in maxilla and mandible between and within the two groups.

**Table 3:** Comparison of the number of teeth present in oral cavity among dentists and laypersons

**Variables**	**Dentists (n = 195)**			**Laypersons (n = 277)**			p-value^*^
	**Male**	**Female**	**p-value**^**a**^	**Male**	**Female**	**p-value**^**b**^	
	**(n = 71)**	**(n = 124)**		**(n = 199)**	**(n = 78)**		
No. of teeth present in maxilla	13.90(1.8)	13.19(1.9)	0.011	12.80(2.0)	11.68(2.3)	<0.0001	
	**13.45(1.9)**			**12.49(2.1)**			**<0.0001**
No. of teeth present in mandible	14.31(1.5)	13.68(1.4)	0.006	13.16(2.1)	12.23(1.9)	0.001	
	**13.91(1.5)**			**12.90(2.1)**			**<0.0001**
Total number of teeth present	28.21(2.9)	26.86(3.0)	0.003	25.96(3.7)	23.91(3.8)	<0.0001	
	**27.35(3.0)**			**25.38(3.9)**			**<0.0001**

Data are expressed in mean (SD).

Bold numbers denote total mean (SD) and p-value among dentists and laypersons.

p-value^a^: *t*-test for difference in mean between males and females among dentists.

p-value^b^ : t-test for differences in mean between males and females among laypersons.

p-value*: t-test for differences in mean among dentists and laypersons.

The self-reported oral health behavior is presented in Table 4. The frequency of tooth brushing was higher among the dentists with 76% of male and 90% of female dentists brushing their teeth twice a day or more. Brushing teeth once a day was more common among laypersons (94% male and 92% female). Almost 90% of the dentists reported to always use toothpaste containing fluoride while brushing their teeth compared to 29% of laypersons (Table 4).

**Table 4:** Comparison of the self-reported oral health behavior among the dentists and laypersons

**Variables**	**Dentists (n = 195)**			**Laypersons (n = 277)**		
	**Male**	**Female**	**p-value**^**a**^	**Male**	**Female**	**p-value**^**b**^
	**(n = 71)**	**(n = 124)**		**(n = 199)**	**(n = 78)**	
**Brushing habits**						
Twice a day or more	76.1	89.5	0.012			
Once a day or less	23.9	10.5		93.9	92.3	0.628
Not daily (laypersons)				6.1	7.7	
**Interdental space cleaning**						
Twice a day or more	19.7	18.9	0.068	29.8	33.3	0.746
Once a day or less	71.8	79.5		62.6	57.7	
Never	8.5	1.6		7.6	9.0	
**Using fluoridated toothpaste**						
Always or almost always	94.3	87.1	0.114	26.6	33.8	0.237
Seldom	5.7	12.9		73.4	66.2	

Data are expressed in percentage.

p-value^a^ : *χ*^2^ test for differences between males and females among dentists.

p-value^b^ : *χ*^2^ test for differences between males and females among laypersons.

Regarding the self-reported satisfaction about their present oral health status, above 95% of participants from both groups reported their condition of teeth and mouth to be good. About 94% of dentists emphasized the maintenance of their oral health to be very important compared to 19% of laypersons (p < 0.0001). Similarly, a significant difference (p < 0.0001) was observed regarding personal satisfaction with the appearance (73% dentists versus 55% laypersons) and function (88% dentists versus 55% laypersons) of teeth (Table 5). Almost 42% of laypersons reported that they had never visited a dentist previously compared to 1.5% of the dentists. The majority (64%) of the laypersons who had visited a dentist did so for treatment due to symptoms rather than for a regular checkup (data not shown).

**Table 5:** Comparison of the self-reported condition, appearance, function and maintenance of teeth among the dentists and laypersons

**Variables**	**Dentist**	**Laypersons**	**p-value**
	**(n = 195)**	**(n = 277)**	
**Maintaining teeth**			
Quite important	5.7	6.1	<0.0001
Very important	94.3	19.1	
Average	-	74.7	
**Appearance of teeth**			
Satisfied	72.7	55.1*	<0.0001
Neither/ nor	22.2	39.5	
Dissatisfied	5.2	5.4	
**Function of teeth**			
Satisfied	87.6	54.7	<0.0001
Neither/ nor	9.8	39.1	
Dissatisfied	2.6	6.2	
**Condition of teeth and mouth**			
Good	96.9	96.0	0.605
Bad	3.1	4.0	

Data are expressed in percentage.

p-value: *χ*^2^ test for differences between dentist and laypersons.

*Significant difference was seen between gender (male- 60% and female- 42%) in laypersons (p = 0.008).

## Discussion

In this study we found that dentists have better oral health behavior and oral health status compared to general population. Most of the dentists brush their teeth at least twice daily using fluoridated toothpaste and clean interdental space at least once daily. The proportion of decayed teeth was significantly lower among dentists compared to laypersons. Total number of teeth present in the oral cavity was also comparatively higher among dentists, although there was no significant difference in the total DMFT score between the groups. Periodontal status among dentists (CPITN score 0) was also better than among the laypersons that had significantly more participants with CPITN score of 3 with self-reported tooth mobility and gingival bleeding.

Oral health behavior of a person is very important for oral disease prevention and is determined by the brushing habits, interdental space cleaning and regular dental visits [15]. Education contributes to improved knowledge, and as the level of education increases, there is improvement in the level of oral health awareness, attitude and behavior [16,17]. Only 38% of laypersons in our study had at least bachelor level education. Therefore, our finding of dentists having better oral health behavior and oral health status compared to general population is not unexpected as it is likely to be related to their education, training and better awareness towards oral health care. On the other hand, as this self-reported better oral health behavior among dentists was not associated with lower caries prevalence, the possibility of over reporting of good dental behavior by the dentists cannot be excluded.

The reason behind lower prevalence of decayed teeth and higher prevalence of restored teeth among dentists compared to laypersons could be due to better oral hygiene or more frequent dental visits, better access to care and treatment when indicated. However, as the proportion of caries-free subjects was similar in both groups, over-treatment among dentists cannot be excluded.

The majority of laypersons reported brushing their teeth once a day only and very few used fluoridated toothpaste even though they were readily available in the market. One of the reasons behind this could be lack of awareness regarding the use of fluoridated toothpastes [18,19]; as most laypersons seem to have not adequate knowledge about fluoridated toothpaste and its benefits [20]. Our study has also confirmed that brushing once daily is still a common practice among people in Nepal which was also reported previously by the ‘National Pathfinder Survey’ [21]. Considering these results, it seems important to make efforts and take measures to inform general population about the advantages of using fluoridated toothpaste and brushing teeth twice daily.

Most people in Nepal take snacks in between main meals [22] and have a tradition of drinking tea/coffee with sugar at least twice daily, early in the morning and in the afternoon. Nepalese dentists also reported taking sugar containing food in between the main meals despite the knowledge that the diet rich in sugar is one of the main factors in the etiology of caries [23]. Similar habits were reported previously in 49% of Mongolian dentists [11] and 60% of Iranian senior dental students [24] who preferred sugar-containing food between the main meals.

Mongolian dentists of similar age group (23-60 years) had less number of total teeth present (mean 24.9) and higher mean DMFT (6.4) compared to their Nepalese dentists’ counterparts. Similarly, the mean numbers of FT (3.2) and MT (2.9) were also higher but DT (0.3) was much lower in Mongolian dentists compared to what we found among Nepalese dentists [11]. However, the distribution pattern of the caries was similar, molars followed by the premolars, as reported in previous studies [23].

Although decayed and unfilled teeth were more frequent among Nepalese laypersons compared to dentists, the total number of teeth present in the oral cavity (25.4) was similar to that of the Mongolian dentists (24.9), and their mean DMFT (4.4) was lower compared to a group of adult population in India (5.1) and Australia (16.6) [11,25,26]. These differences could be related to dietary habits, education level, socio-economic status and access to oral health care [27].

Earlier studies from Nepal have reported 93-100% of the 33-40 year olds having calculus and 3-34% having deep periodontal pockets [28]. Corbet et al. reported 80-100% of Asian adults having calculus or experiencing deep periodontal pockets in spite performing daily oral hygiene practice [29]. In line with this, more than half of laypersons reported gingival bleeding and tooth mobility in our study, and majority of them had CPITN score of 3. Periodontal status was much better among dentists in our study indicating that knowledge and awareness about oral hygiene and preventive dental care are important factors for better oral health status. Although more than half of the laypersons reported to have visited dentists previously, the frequency of visit in a period of two years was low and the reason for the visit was mostly for treatment rather than regular checkup. It demonstrates the lack of awareness on importance of regular dental visits for maintaining good oral health among the laypersons. Dental visits were reported to be mostly for curative purpose also in Mongolia [11]. Factors, such as person’s level of education, income, knowledge and attitude towards oral health and oral health care personnel may affect frequency of visits to dental services [30,31].

There are a few published studies that report the oral health behavior or oral health status among dentists in USA [32], Iran [33,34], Korea [7] and Mongolia [11] but so far, no such study has been reported among Nepalese dentists. In our study, we included only the dentists practicing in Kathmandu valley and therefore the generalisability of the results for other dentists may be questioned. Nonetheless, since most dentists practice in the capital city, the sample population is likely to be representative of dentists working in Nepal. Laypersons were randomly selected from a population of “healthy” adults who were accompanying a relative or a friend in the hospital, therefore possibility of sampling bias cannot be excluded. The male/female ratio among the participating dentists was 1:1.7 and 2.5:1 among laypersons, which indicates possibility of selection bias, could have influenced our results. However, a similar trend of higher female dentist (80%) participation was seen in Mongolia [11], whereas an opposite trend has been reported in a study from Korea with 91% male participation [7]. A higher male to female participation ratio among laypersons in our study could be because males usually accompany the sick patient to the hospital.

This study was carried out in 2006 and some changes in the provision of oral health care may have taken place during the last few years. Dental education in Nepal started in the year 1997 and two private dental institutes started a Bachelor of Dental Surgery (BDS) program. In the end of 2008, the total number of registered dentists or dental surgeons was 756 with 49 dental specialists in various fields and 5 dental colleges are established so far [35]. Despite an increase in number of dentists and doctors [36,37] in the country, there is a still a shortage of dental care service in the rural areas. According to WHO oral health Country/Area Profile Programme (CAPP), the ratio of dentist per inhabitant in Nepal is 1:47306 [38].

The oral health status reported in this study has been based on clinical examination performed by a trained dentist. However, the dental caries was recorded by visual and tactile method without taking radiographs. The oral health behavior was self-reported; hence chances of bias due to over- or underreporting of information cannot be excluded [39].

Finally, although the questionnaire has been previously validated in a comparable population [11] it was not validated in the target Nepalese population. Therefore any variations in published results should be interpreted with caution.

## Conclusions

Our study has shown that dentists have better self-reported oral health behaviors and periodontal status than the laypersons. Despite no significant difference in caries prevalence between the two groups, the prevalence of decayed and unfilled teeth was lower among dentists. Since dental caries and periodontal diseases can be prevented with effective oral hygiene practices, educating public to improve oral health awareness and develop effective oral-care habits should be considered.

## Abbreviations

CAPP: Country/Area Profile Program; CPI: Community Periodontal Index; CPITN: Community Periodontal Index for Treatment Needs; DMFT: Decayed Missing Filled teeth; DT: Decayed tooth; FT: Filled tooth; MT: Missing tooth; NDA: Nepal Dental Association; NHRC: Nepal Health Research Council; WHO: World Health Organization.

## Competing interests

The authors declare that they have no competing interests.

## Authors’ contributions

Study concept, design and methodology – MW, TAT. Data collection and data entry- MW. Supervision - TAT. Analysis and interpretation of data - MW, TAT, PB, GA. Writing, review, critique, comments and revision of manuscript - MW, TAT, PB, GA. All authors read and approved the manuscript.

## Pre-publication history

The pre-publication history for this paper can be accessed here:

http://www.biomedcentral.com/1472-6831/14/23/prepub
